# Succession of Cyanobacterial Community Contributes to Bacterial and Fungal Community Assembly in Dryland Biocrusts

**DOI:** 10.1002/ece3.73151

**Published:** 2026-03-02

**Authors:** Kang Zhao, Ran Zhao, Khan Ajmal, Wei Chen, Qiuping Zhang, Bingchang Zhang, Fei Wang

**Affiliations:** ^1^ School of Life Sciences Shanxi Normal University Taiyuan China; ^2^ Research Center for Ecological Restoration, School of Life Sciences Shanxi Normal University Taiyuan China; ^3^ Department of Environmental Sciences Kohat University of Science and Technology Kohat Pakistan; ^4^ Biodiversity and Ecological Function Research Group of Middle Reaches of Yellow River, Geographical Science College Shanxi Normal University Taiyuan China

**Keywords:** cyanobacteria, keystone taxa, microbial co‐occurrence networks, photoautotrophs, succession of biocrusts

## Abstract

Biological soil crusts (Biocrusts), which are widely distributed across arid and semi‐arid surfaces, play important ecological roles. Cyanobacteria are considered key intrinsic drivers of biocrust persistence and functioning, exerting a profound influence on their ecological roles. Although the distribution patterns and environmental drivers of cyanobacteria have been extensively studied in biocrusts, their role in microbial community assembly remains insufficiently understood. This study investigated the dynamics of cyanobacterial communities during biocrust succession and their relationships with bacterial and fungal community variations. The results revealed pronounced shifts in the cyanobacterial community, explained by ASV (amplicon sequence variants) turnover and changes in dominant taxa such as Microcoleaceae, unclassified Cyanobacteriales, and Chroococcidiopsidaceae. Total phosphorus, nitrogen, and pH were identified as key environmental factors associated with changes in cyanobacterial community. The bacterial community was primarily governed by homogeneous selection within deterministic processes, whereas the fungal community appeared to be shaped by stochastic processes and variable selection within deterministic processes. Together with abiotic factors such as phosphorus, nitrogen, soil organic carbon, and pH, the cyanobacterial community significantly contributed to bacterial and fungal community structure, as supported by multiple analytical approaches. A few cyanobacterial species from Chroococcidiopsidaceae, Microcoleaceae, and Nostocaceae were identified as keystone taxa in the microbial co‐occurrence network, enhancing its stability during early biocrust development. These keystone cyanobacteria also underwent succession and exhibited strong co‐occurrence with specific microorganisms, including *Craurococcus caldovatus*, *Rubellimicrobium*, *Rubrobacter*, and *Microvirga*. Overall, these findings elucidate how cyanobacteria are involved in structuring microbial communities during biocrust succession and provide a theoretical basis for improving biocrust restoration in dryland ecosystems.

## Graphical abstract

Cyanobacteria are key components driving the formation and development of biological soil crusts in dryland ecosystems. Their community distribution differs significantly across stages of biocrust development. The presence of cyanobacteria influences variations in bacterial and fungal communities within biological soil crusts by regulating the physicochemical properties and potential interspecific microbial interactions.

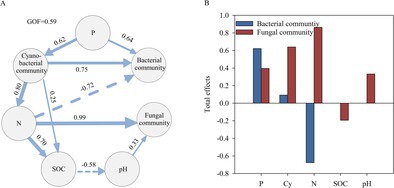

## 1 Introduction

Biological soil crusts (biocrusts) are essential for ecological restoration in dryland ecosystems. As a typical surface cover in dryland soil, biocrusts occupy approximately 12% of the global terrestrial area and 13.9% of China's drylands (Rodriguez‐Caballero et al. 2018; Qiu et al. 2023). These organic complexes consist of photoautotrophs (e.g., cyanobacteria, algae, lichens, and bryophytes), heterotrophs (e.g., bacteria, fungi, and archaea), and soil particles (Weber et al. 2022). They play vital ecological roles in enhancing soil fertility, facilitating biogeochemical cycling, preventing soil erosion, and supporting vegetation succession (Belnap 2003; Eldridge et al. 2020).

Cyanobacteria are key functional components of biocrusts, driving their development and influencing their ecological functions. Research on their distribution in biocrusts and responses to environmental changes has been extensive in recent decades. Cyanobacteria are thought to rely on biocrust habitats and contribute to biocrust development through their functions (Büdel et al. 2016). A typical variation feature of the cyanobacterial community is the change from *Microcoleus* and *Schizothrix* in bare sand to *Scytonema*, *Tolypothrix*, and *Nostoc* in well‐developed biocrusts (Garcia‐Pichel and Wojciechowski 2009; Gao and Garcia‐Pichel 2011). Key factors affecting their distribution in biocrusts include temperature and moisture, such as mean annual precipitation and temperature (Zhang et al. 2016; Zhao et al. 2018; Angeles Munoz‐Martin et al. 2019). Changes in cyanobacterial communities are closely linked to the development and ecological functions of biocrusts.

Cyanobacteria form the physical structure of biocrusts (Garcia‐Pichel and Wojciechowski 2009). Filamentous cyanobacteria, in particular, are the main agents used in artificial biocrust construction (Zhou et al. 2020; Rossi et al. 2022). Moreover, their key ecological functions—carbon and nitrogen fixation—are essential for maintaining microbial community structure and function in oligotrophic desert soils (Yeager et al. 2007; Munoz‐Rojas et al. 2018). The fixed carbon and nitrogen are believed to stimulate heterotrophic metabolism, driving nutrient transformations and shaping heterotrophic microbial composition in biocrusts (Maier et al. 2018; Fisher et al. 2020). Despite their importance, the role of cyanobacteria in microbial community assembly within biocrusts remains poorly understood.

The microbial community, essential for the multifunctionality of terrestrial ecosystems (Bardgett and van der Putten 2014; Delgado‐Baquerizo et al. 2016), is shaped by both deterministic processes (environmental filtering and interspecific interactions) and stochastic processes (random birth, death, and dispersal) during assembly (Stegen et al. 2013; Zhou and Ning 2017). Previous research on biocrust microbial communities has mainly focused on variational patterns (Lan et al. 2017; Maier et al. 2018) and their environmental drivers (Su et al. 2020; Ohan et al. 2023). For instance, nitrogen, organic carbon, and phosphorus content have been identified as key factors influencing bacterial and fungal community variations (Zhang et al. 2016; Miralles et al. 2020; Su et al. 2020). However, the microbial community assembly process in biocrusts has not been systematically analyzed from the perspective of interspecies interactions from specific functional groups.

Microorganisms form intricate interaction networks rather than existing in isolation (Faust and Raes 2012). Interspecific interactions are key regulators of community assembly, alongside deterministic processes such as environmental filtering and stochastic events including random birth, death, and dispersal (Stegen et al. 2013; Zhou and Ning 2017). Microbes interact through resource competition, metabolic mutualism, or antagonism (Zhao et al. 2023), and such relationships are common within biocrusts. For instance, lichenized fungi and Chlorophyta symbioses were thought to play crucial roles in shaping biocrust microbial communities (Xu et al. 2021). However, direct examination of pairwise microbial interactions remains challenging due to cultivation difficulties and analytical complexity. Co‐occurrence network analysis provides an effective alternative for inferring potential microbial relationships (Barberán et al. 2012; Röttjers and Faust 2018). Although co‐occurrence networks cannot precisely identify species interactions (Blanchet et al. 2020; Neu et al. 2021), they reveal potential coexistence patterns across taxa and ecosystems (Eiler et al. 2012; Li et al. 2021).

This study investigated cyanobacterial community dynamics during biocrust succession in the Gurbantunggut Desert and their associations with bacterial and fungal community variations. We aimed to elucidate the assembly mechanisms of bacteria and fungi from the perspective of cyanobacteria‐related interactions. Considering the prominent carbon and nitrogen fixation capacities of cyanobacteria in desert biocrusts (Belnap 2002; Munoz‐Rojas et al. 2018), we hypothesized that a few abiotic properties correspond with cyanobacterial distribution. Cyanobacteria‐associated microbial coexistence contributes to variations in bacterial and fungal communities. To test this hypothesis, we analyzed bacterial, fungal, and cyanobacterial community variations and related physicochemical properties across biocrust developmental stages and explored potential microbial associations using co‐occurrence networks.

## 2 Materials and Methods

### 2.1 Study Area and Sampling

The study was conducted in the Gurbantunggut Desert, a typical fixed to semi‐fixed temperate desert with mean annual precipitation of 80–160 mm and a temperature of 5.0°C–5.7°C (Zhang et al. 2007). The landscape and vegetation, dominated by *Haloxylon ammodendron* and *H. persicum* (< 30% cover), have been described previously (Zhou et al. 2020). Lichen and moss crusts are widespread, while bare sand and nascent algal crusts occur due to wind erosion and human disturbance. As detailed previously (Zhao et al. 2024), samples were collected in June 2021 from nine interdune sites spaced at least 1 km apart (44°15′15.75″ N, 87°40′27.55″ E, Figure S1). Bare sand and biocrusts (algal, lichen, and moss types, ~2 cm thick) were visually identified and collected, yielding 36 samples (nine replicates per stage). Samples were stored at −80°C for DNA extraction and physicochemical analysis.

### 2.2 Physicochemical Properties

Physicochemical properties were analyzed using standard methods described in the previous study (Zhao et al. 2024). The detailed measurement procedures and data are shown in Appendix S1 and Table S1. Briefly, the nitrate, ammonium, total nitrogen (TN), total phosphorus (TP), available phosphorus (AP), soil organic carbon (SOC), pH, and moisture of different biocrust developmental stages were measured.

### 2.3 DNA Extraction and Amplicon Sequencing

Genomic DNA was extracted from 0.25 g of each sample using the SPINeasy DNA Kit for Soil (MP Biomedicals, USA). DNA quality and concentration were assessed with a Nanodrop ND‐2000 spectrophotometer (Thermo Scientific, USA). A detailed description of 16S rDNA and ITS amplicon sequencing and bioinformatics analysis can be found in Appendix S2, which follows a widely used sequencing strategy. Briefly, the bacterial 16S rDNA gene and the internal transcribed spacer (ITS2) region of the fungi were used to identify the bacterial and fungal communities in biocrusts. The primer set was 515F and 806R for 16S rDNA (Caporaso et al. 2011), and gITS7 and ITS4 for ITS (Ihrmark et al. 2012). The paired‐end Illumina sequencing strategy was used based on the MiSeq platform (Biomarker Technologies Co. Ltd., Beijing, China). Sequence data are available at NCBI under accession numbers PRJNA1048298 (16S) and PRJNA1048333 (ITS).

### 2.4 Cyanobacterial Community Analysis

The 16S rDNA amplicon sequencing data identified the cyanobacterial community by assigning phylogenetic information to the represented ASVs (Amplicon Sequence Variants). To enhance the accuracy of cyanobacterial phylogenetic classification, the identified cyanobacteria were taxonomically reclassified using the NCBI Taxonomy Browser (https://www.ncbi.nlm.nih.gov/Taxonomy/Browser/wwwtax.cgi), based on the taxa identified with the SILVA (v138.1) database. To minimize the influence of varying sequencing depths of the samples, the cyanobacterial sequences were rarefied to 1648 reads.

### 2.5 Microbial Community Assembly

Community assembly processes were evaluated using a null‐model‐based framework (Stegen et al. 2013; Jiao et al. 2022). In brief, an inferred neighbor‐joining phylogenetic tree was constructed, and null‐model‐based phylogenetic β‐diversity metrics (βNTI) were employed to assess disparities in phylogenetic diversity. The community assembly process was categorized according to the βNTI. The contributions of homogeneous selection (βNTI < −2, indicating lower‐than‐expected phylogenetic turnover, with microbial communities being more phylogenetically similar than anticipated), variable selection (βNTI > 2, indicating greater‐than‐expected phylogenetic turnover, with microbial communities exhibiting higher phylogenetic dissimilarity than predicted), and the stochastic process (|βNTI| < 2, indicating random changes in community structure) to the community assembly were calculated based on the probability of βNTI occurrence in different biocrust successional stages.

### 2.6 Co‐Occurrence Network Analysis

Microbial co‐occurrence networks were constructed using the IDENs pipeline (Feng et al. 2022) based on SparCC correlations (*r* ≥ 0.65, *p* ≤ 0.05). Bacterial ASVs and fungal OTUs (operational taxonomic units) with relative abundance > 0.1% and present in ≥ 7 replicates per stage were included. Two networks—with and without cyanobacterial nodes—were compared to assess cyanobacterial effects on network stability. Network topological properties, robustness, and vulnerability were calculated, with robustness estimated under random (50%) and targeted node removal (100 permutations) (Yuan et al. 2021). Keystone taxa were identified using within‐module (Zi) and among‐module (Pi) connectivity metrics, including connectors (zi ≤ 2.5, Pi > 0.62), module hubs (zi > 2.5, Pi ≤ 0.62), and network hubs (zi > 2.5, Pi > 0.62) (Deng et al. 2012). Subnetworks related to cyanobacteria were visualized in Gephi (v0.9.7).

### 2.7 Statistical Analysis

All analyses were performed in R (version 4.2.0) (R Core 2014). Alpha diversity (Shannon and ACE indices) was computed at the operational taxonomic unit (OTU) or amplicon sequence variant (ASV) level using the vegan package (Dixon 2003). Differences were assessed via One‐Way ANOVA. The discrepancies in bacterial (without cyanobacteria), fungal, and cyanobacterial community were ascertained through nonmetric multidimensional scaling (NMDS), analysis of similarities (ANOSIM), and permutational multivariate analysis of variance (PERMANOVA) in R. Redundancy analysis (RDA) explored relationships among microbial communities and biocrust properties. The individual effects of biocrust properties on microbial community variations were analyzed using the rdacca.hp. package in R based on the RDA (Lai et al. 2022). The NMDS, ANOSIM, and RDA were all based on the OTU/ASV tables.

Partial Least Squares Path Modeling (PLSPM) was used to evaluate the direct and indirect effects of biocrust properties on microbial communities using the *plspm* package (Sanchez 2013). The original model was constructed on the hypothesis that environmental variables play roles in structuring the microbial community. Additionally, the impact of cyanobacteria on carbon and nitrogen accumulation in desert soils was considered as previous studies suggested (Munoz‐Rojas et al. 2018; Lan et al. 2021). Edaphic variables were grouped into pH, soil organic carbon (SOC), nitrogen (TN and ammonium), and phosphorus (TP and AP). The first two PCoA axes represented cyanobacterial and fungal communities, while bacterial (without cyanobacteria) diversity was represented by the Shannon index.

## 3 Results

### 3.1 Diversity and Composition of Cyanobacterial Community

**FIGURE 1:**
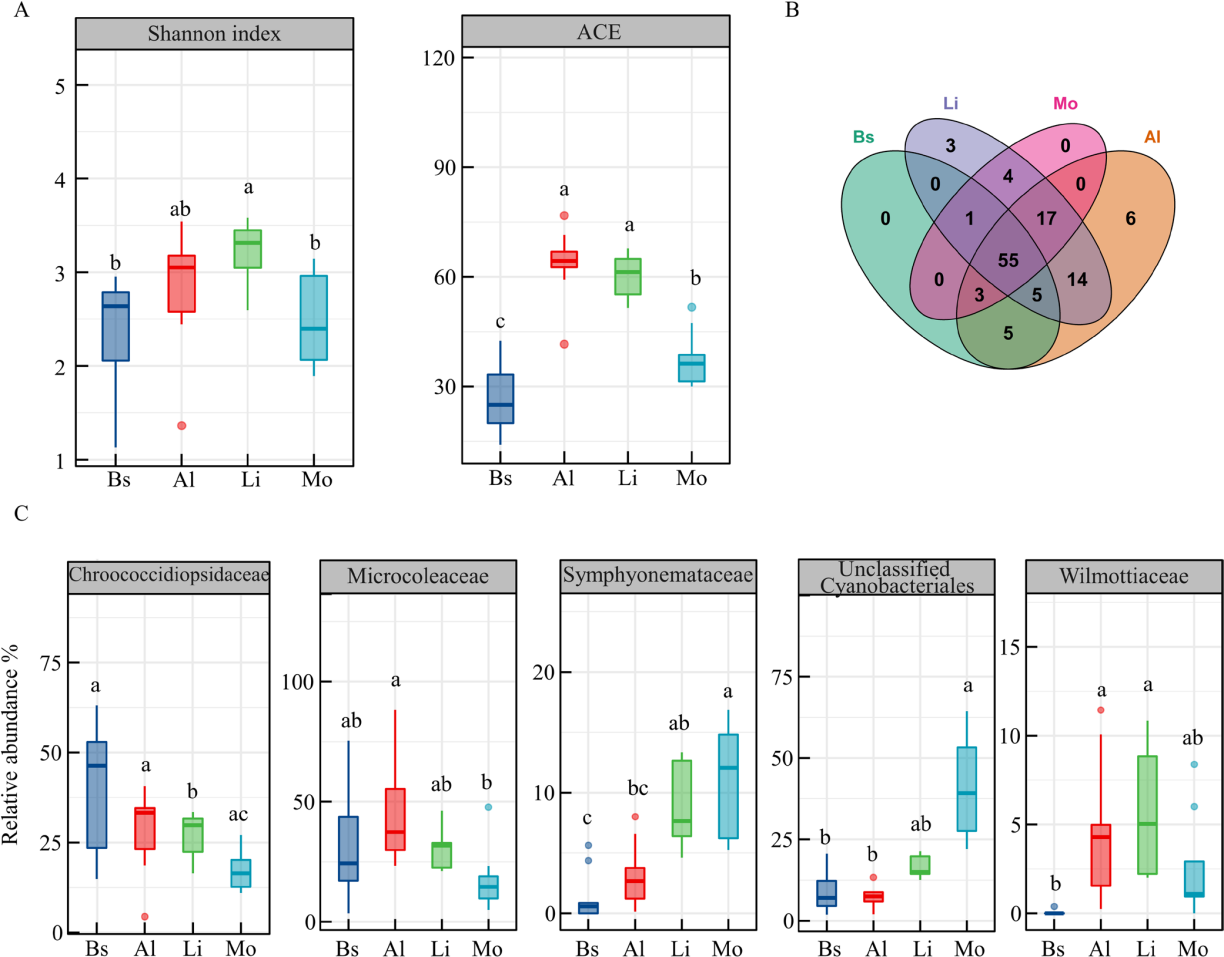
Alpha diversity (A), ASV distribution (B), and community composition (C) of cyanobacteria with biocrust succession. Al, algal crust; Bs, bare sand; Li, lichen crust; Mo, moss crust. Different lowercase letters indicate significant differences (*p* < 0.05).

Cyanobacterial diversity varied markedly across biocrust succession (Figure 1A). The Shannon index increased from bare sand to algal and lichen crusts, then declined in the moss crust. The highest ACE index was observed in the algal crust, followed by the lichen crust. A total of 113 cyanobacterial ASVs were identified, with 55 shared across all crust types, representing 33%–53% of total sequences (Figure 1B). Six ASVs were unique to algal crusts (3.1%) and three to lichen crusts (0.3%). Twelve families were detected, dominated by Chroococcidiopsidaceae, Microcoleaceae, Symphyonemataceae, Wilmottiaceae, and unclassified Cyanobacteriales (> 5% relative abundance, Figure 1C). Lower‐abundance families included Gomontiellaceae, Coleofasciculaceae, Nostocaceae, and Leptolyngbyaceae (Figure S2). The most abundant genera were *Tychonema* and *Crinalium* (Figure S3).

**FIGURE 2:**
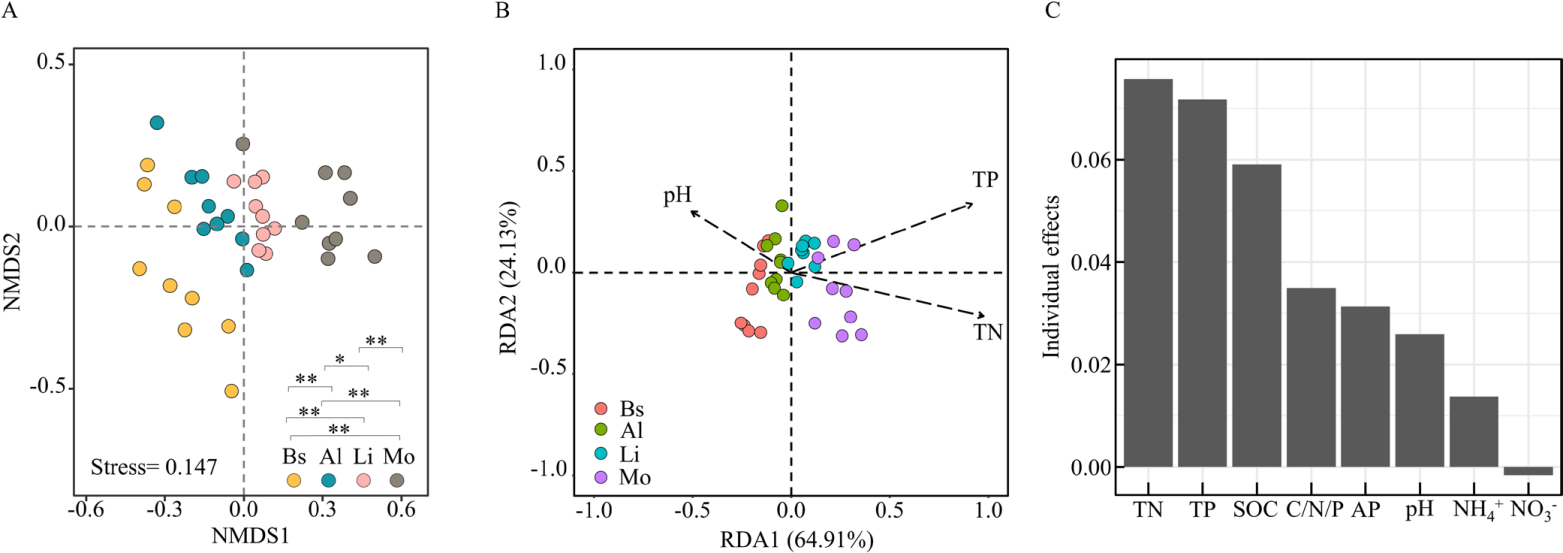
Variational patterns of cyanobacterial community and associated edaphic factors with biocrust succession. (A) NMDS of cyanobacterial structure. Differences among successional stages were tested using PERMANOVA. (B) RDA of cyanobacterial community in response to edaphic factors. Due to the collinearity between SOC and TN, SOC was excluded. (C) Individual effects of edaphic factors on cyanobacterial community. Al, algal crust; AP, available phosphorus; Bs, bare sand; Li, lichen crust; Mo, moss crust; NH^4^+, ammonium; NO^3^−, nitrate; SOC, soil organic carbon; TN, total nitrogen; TP, total phosphorus. *p < 0.05, **p < 0.01.

Nonmetric multidimensional scaling (NMDS) and PERMANOVA analyses confirmed distinct cyanobacterial community structures across developmental stages (Figure 2A). Redundancy analysis (RDA) identified total phosphorus (TP), total nitrogen (TN), and pH as primary environmental predictors (Figure 2B). These factors also exhibited strong individual effects on the variation in cyanobacterial community (Figure 2C). Most cyanobacterial families correlated positively with TP, TN, ammonium, and SOC content except Chroococcidiopsidaceae and Gomontiellaceae. Soil pH negatively correlated with Nostocaceae and unclassified cyanobacteriales, but positively correlated with Microcoleaceae (Figure S4).

### 3.2 Diversity and Assembly Processes of Microbial Communities

**FIGURE 3:**
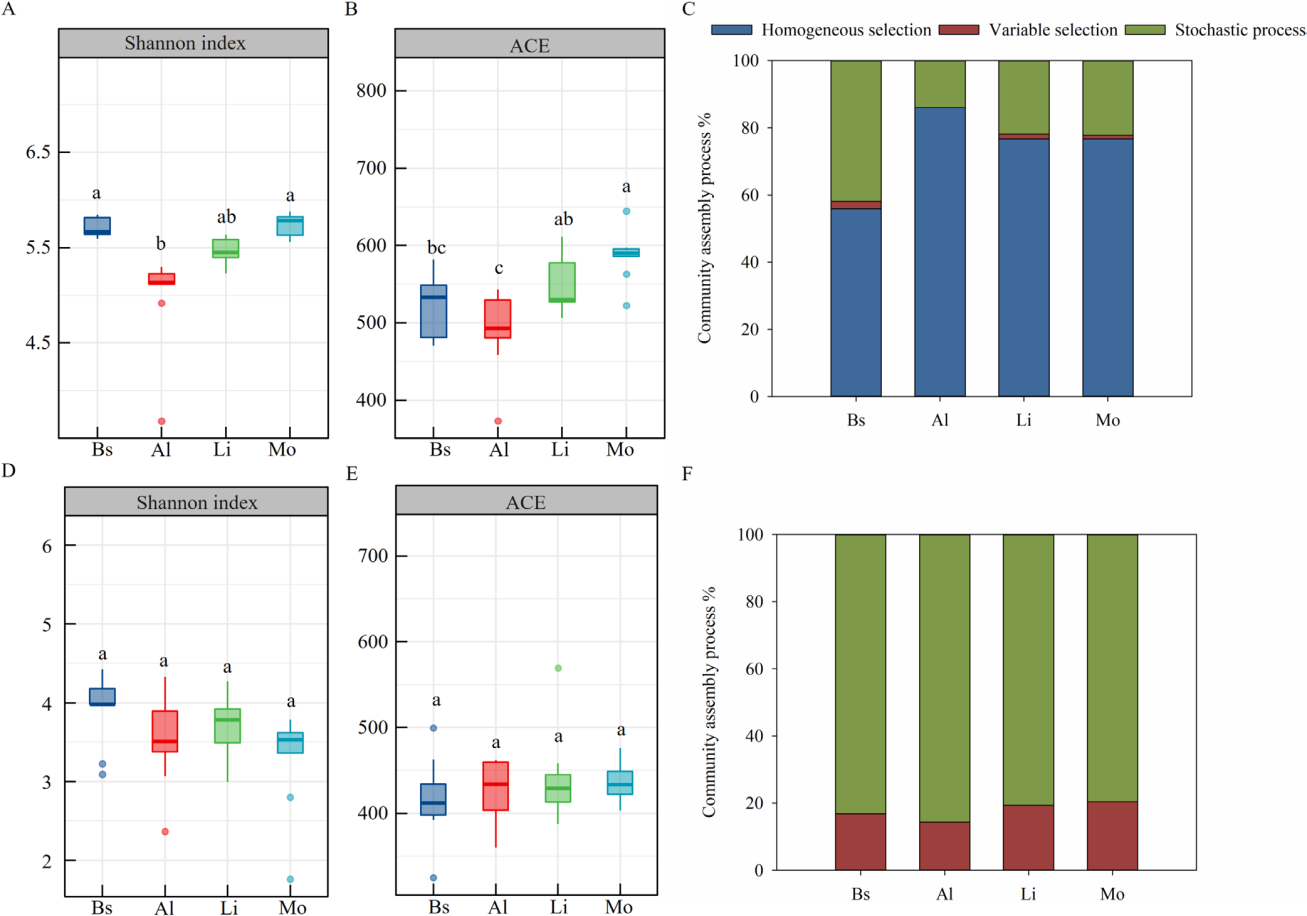
Alpha diversity and community assembly processes of bacterial (A–C) and fungal (D–F) communities across different biocrust successional stages. The bacterial community index was calculated based on the ASV table without cyanobacteria. Al, algal crust; Bs, bare sand; Li, lichen crust; Mo, moss crust. Different lowercase letters indicate significant differences (*p* < 0.05).

Both bacterial and fungal communities exhibited distinct successional patterns (Figure 3). The bacterial Shannon index decreased in algal crusts but increased in lichen and moss crusts, while abundance‐based coverage estimator (ACE) generally increased throughout the succession process. In contrast, fungal diversity declined gradually, with ACE remaining stable. The community assembly processes of bacteria and fungi differed notably. Bacterial community assembly was primarily predicted by homogeneous selection (56%–86%), a deterministic process (Figure 3C). In contrast, fungal community assembly was mainly governed by stochastic processes, followed by variable selection of a deterministic process (14%–20%) (Figure 3F).

### 3.3 Variations in Bacterial and Fungal Communities and Associated Biocrust Properties

Taxonomic analysis identified three dominant bacterial classes: Alphaproteobacteria (24.70%), Cyanobacteria (24.08%), and Actinobacteria (21.96%) (Table S2). The major fungal classes included unidentified Ascomycota (33.37%), Dothideomycetes (29.50%), unclassified fungi (14.29%), Lecanoromycetes (6.85%), and Eurotiomycetes (6.31%). The dominant bacterial genera were *Craurococcus caldovatus*, unclassified Sphingomonadaceae and Beijerinckiaceae, and *Rubrobacter* (Figure S5A). *Circinaria*, *Alternaria*, and *Comoclathris* were dominant among the fungal community (Figure S5B). The relative abundances of these microbial groups varied significantly across different stages of biocrusts.

**FIGURE 4:**
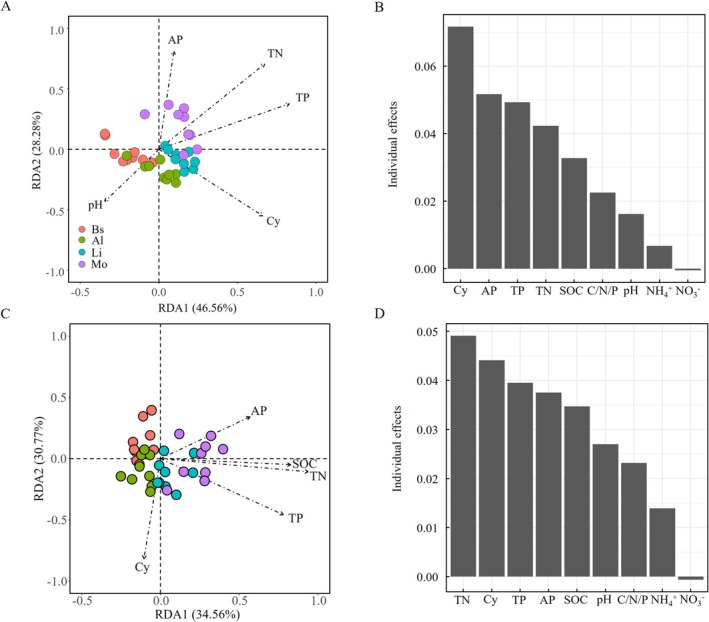
Changes in bacterial and fungal community structures with biocrust succession. Redundancy analysis (RDA) of bacterial (A) and fungal (C) community structures and individual effects of edaphic factors and cyanobacterial communities on bacterial (B) and fungal (D) community variation are shown. The bacterial structure refers to the residual community after removing cyanobacteria. Due to the collinearity between SOC and TN, SOC was excluded from the RDA. Al, algal crust; AP, available phosphorus; Bs, bare sand; Li, lichen crust; Mo, moss crust; NH^4^+, ammonium; NO^3^−, nitrate, SOC, soil organic carbon; TN, total nitrogen; TP, total phosphorus, The C/N/P ratio was calculated as SOC/TN/TP. Cy: Cyanobacterial Shannon diversity.

The distribution patterns of bacterial (without cyanobacteria) and fungal communities were closely associated with the developmental stages of biocrusts (Figure S6 and Table S3). NMDS and ANOSIM confirmed distinct bacterial and fungal structures among biocrust stages (Figure S6C,D). The PERMANOVA provided statistics that bacterial and fungal community compositions differed significantly among the developmental stages (Table S3). Key properties associated with bacterial community (without cyanobacteria) variation included TN, TP, AP, pH, and the cyanobacterial community (Figure 4A,B). These factors and SOC exhibited strong individual effects on bacterial community variation. Similarly, TN, SOC, TP, AP, pH, and ammonium were significantly associated with the variation in the fungal community (Figure 4C,D).

### 3.4 Cyanobacteria‐Associated Microbial co‐Occurrence Patterns

**FIGURE 5:**
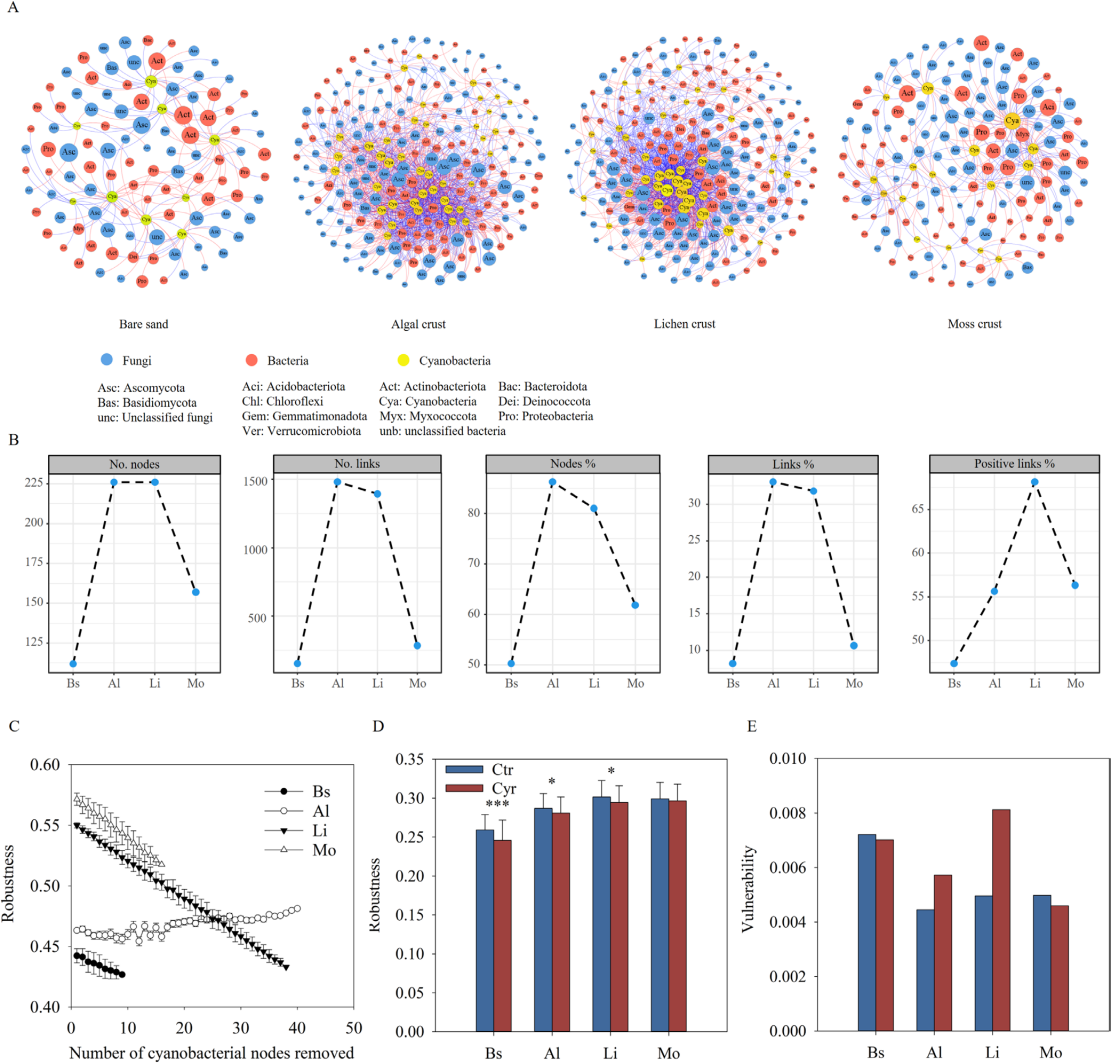
Characteristics of cyanobacteria‐associated microbial subnetworks (A, B) and the impact of cyanobacteria on network stability (C‐E). The trend in network robustness following the random removal of cyanobacterial nodes was shown (C). The robustness (calculated by randomly removing 50% nodes) of co‐occurrence networks (D) and vulnerability (E), with and without cyanobacteria, was compared. Ctr: Network including cyanobacterial nodes; Cyr: Network excluding cyanobacterial nodes. Al, algal crust; Bs, bare sand; Li, lichen crust; Mo, moss crust. *p < 0.05, ***p < 0.001.

Cyanobacteria‐associated subnetworks (Figure 5) were extracted from microbial co‐occurrence networks of biocrusts (Figure S7; Table S4 and Appendix S1). These analyses underscored the vital role of cyanobacteria in maintaining the stability of microbial co‐occurrence networks. Especially in algal and lichen crusts, cyanobacterial nodes accounted for 31.5% and 28.8% of prokaryotic nodes, respectively, and were connected to over 80% of the nodes in the networks (Figure 5B). Most of these co‐occurrences were positive with bacteria and negative with fungi. From bare sand to algal crusts, bacterial correlations shifted from negative to positive, while fungal correlations reversed (Figure S8).

A few cyanobacteria were identified as potential keystone taxa of the microbial co‐occurrence networks (Table S5). These identified potential keystone cyanobacteria were phylogenetically affiliated primarily with Chroococcidiopsidaceae, and fewer with Microcoleaceae and Nostocaceae. Other cyanobacteria were not recognized as being part of the key components in maintaining the microbial co‐occurrence networks. Statistical analysis revealed that the potential keystone Chroococcidiopsidaceae was strongly co‐occurring with *Craurococcus Caldovatus*, *Rubellimicrobium*, *Geodermatophilus*, *Preussia*, and *Alternaria* in the microbial co‐occurrence networks of different biocrusts.

The presence of cyanobacteria stabilized microbial co‐occurrence networks (Figure 5C–E). First, when cyanobacterial nodes were randomly removed, the robustness of the co‐occurrence network declined in most stages of biocrusts (Figure 5C). An exception was observed in algal crusts, where the network remained stable when randomly removing all cyanobacterial nodes. However, removing keystone cyanobacteria significantly decreased robustness in algal crusts (Figure S9). The comparison of these results distinguished node‐identity effects from simple node‐number effects. Additionally, cyanobacteria were more important in maintaining the stability of the microbial community in the early developmental stage of biocrusts. Although most major network parameters did not exhibit substantial changes (Table S4), the complete removal of cyanobacterial nodes significantly reduced the robustness of co‐occurrence networks in early‐stage biocrusts (bare sand, algal, and lichen crusts, Figure 5D) and increased the network vulnerability in algal and lichen crusts (Figure 5E).

### 3.5 Biotic and Abiotic Interactions in Biocrusts

**FIGURE 6:**
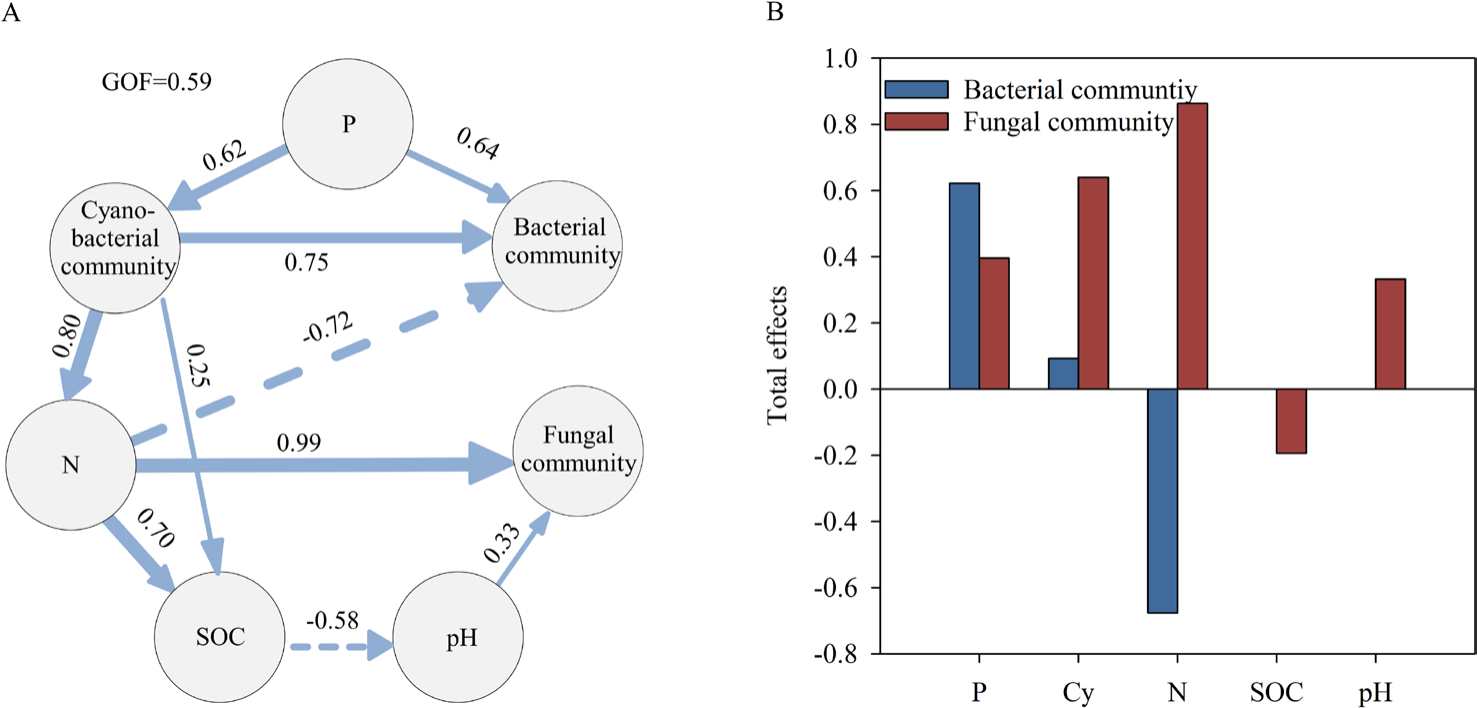
Partial least squares path modeling (PLSPM) illustrating the associations between biotic and abiotic properties of biocrusts. Abiotic factors were grouped into four latent variables: PH, SOC, N (TN and NH^4^+), and P (TP and AP). Cyanobacterial (Cy) and fungal communities were represented by the first two principal coordinates (PCoA), and the Shannon index represented the bacterial community (excluding cyanobacteria). Pseudo‐Goodness of Fit (GoF) was calculated to evaluate model reliability.

As shown in the results above, TN, TP, pH, and SOC were associated with variations in the cyanobacterial community. These environmental factors and the cyanobacterial community had notable effects on the bacterial (without cyanobacteria) and fungal communities. Based on this framework, a partial least squares path modeling (PLSPM) analysis was performed (Figure 6 and Figure S10). The results indicated that phosphorus (TP and AP) was a key factor influencing the structure of the cyanobacterial, bacterial (without cyanobacteria), and fungal communities. It directly or indirectly, through its effects on cyanobacteria, contributed to changes in the non‐cyanobacterial bacterial communities. The phosphorus level also indirectly modulated TN and pH by regulating the cyanobacterial community. These two environmental factors directly affect the fungal community. The total effects of biocrust properties on bacterial and fungal communities highlighted the effects of nitrogen content, followed by the cyanobacterial community, phosphorus (TP and AP), pH, and SOC on the microbial community.

## 4 Discussion

Stable microbial communities underpin the ecological functions of biocrusts, as microorganisms drive microscale processes and regulate ecosystem functioning (Delgado‐Baquerizo et al. 2016). In drylands, biocrusts are critical for soil stabilization and nutrient cycling, and cyanobacterial inoculation is a common restoration strategy (Zhou et al. 2020; Rossi et al. 2022). However, the long‐term persistence of constructed biocrusts was often limited by the low adaptability of inoculated cyanobacteria and the high water demands during the initial phase (Bowker et al. 2020; Zhou et al. 2020). Understanding how cyanobacteria contribute to microbial community structure throughout biocrust succession is therefore critical for advancing both the theoretical understanding and practical application of biocrust restoration.

### 4.1 Phosphorus as a Key Predictor of Cyanobacterial Distribution

Cyanobacteria in dryland biocrusts have been extensively investigated over the past few decades (Garcia‐Pichel et al. 2001; Munoz‐Rojas et al. 2018; Angeles Munoz‐Martin et al. 2019). Some taxa, such as *Microcoleus* and *Nostoc*, are globally distributed (Büdel et al. 2016). Due to the limitations of species‐level identification, taxa in this study were resolved at the family level, consistent with previous microscopic observations from the same region (Zhang et al. 2009, 2016). The dominant cyanobacterial families—Chroococcidiopsidaceae and Microcoleaceae—were also prevalent in Mediterranean ecosystems, the northwestern Negev Desert, and Brazil (Hagemann et al. 2015; Munoz‐Rojas et al. 2018; de Lima et al. 2021). Other major families, including Symphyonemataceae, Wilmottiaceae, Nostocaceae, and Coleofasciculaceae, were widely detected in different regions (Angeles Munoz‐Martin et al. 2019; Wang et al. 2020).

Cyanobacterial community structure varied markedly with biocrust development. On the one hand, the variation was derived from substantial changes in species composition across different stages of biocrust. A total of 113 cyanobacterial ASVs were identified, exceeding previous morphologically based estimates (25–31 species) (Zhang et al. 2009), and the 76 OTUs reported from the Kyzyl Kum and Tengger deserts (Wang et al. 2020). Only 55 ASVs were shared across all four biocrust stages, indicating substantial niche differentiation. On the other hand, the relative abundances of dominant groups such as Microcoleaceae, Symphyonemataceae, and Chroococcidiopsidaceae fluctuated significantly across successional stages. These changes contributed to significant differences in cyanobacterial community structure of different biocrust types.

Total phosphorus (TP) emerged as the primary factor shaping cyanobacterial distribution, with TN, pH, and SOC also showing significant associations. This result was consistent with previous studies in Mediterranean ecosystems, the Chihuahuan Desert, the Great Basin Desert, and Brazil (Angeles Munoz‐Martin et al. 2019; Giraldo‐Silva et al. 2020; de Lima et al. 2021). However, as previous studies suggested, cyanobacteria strongly regulated the nitrogen, organic carbon, and pH of biocrusts. For example, mature biocrusts often harbor abundant nitrogen‐fixing heterocystous taxa, including *Scytonema*, *Tolypothrix*, and *Nostoc* (Gao and Garcia‐Pichel 2011). These heterocystous cyanobacteria are crucial for nitrogen accumulation in late‐stage biocrusts (Belnap 2002; Yeager et al. 2007). Cyanobacteria also contribute to organic matter accumulation in sparsely vegetated arid regions (Büdel et al. 2018; Munoz‐Rojas et al. 2018), leading to pH reduction through the production of organic acids such as acetic, formic, and humic acids (Chapin et al. 2011; Zhao et al. 2018). Therefore, TP was identified as the key predictor of cyanobacterial community variation.

Phosphorus enrichment in biocrusts primarily results from absorption and adsorption by organisms and soil particles (Baumann et al. 2018; Damian et al. 2020), rather than direct biological production. Because phosphorus limitation is widespread and exacerbated by competition among plants and microorganisms (Yang and Post 2011; Stutter et al. 2012), positive correlations between most cyanobacterial groups and TP or AP in this study likely reflect adaptation to phosphorus scarcity. Consequently, low phosphorus availability may restrict phosphorus‐dependent taxa, thereby shaping cyanobacterial community structure.

### 4.2 Abiotic and Cyanobacterial Effects on Microbial Community

Deterministic processes dominated bacterial community assembly, primarily through homogeneous selection, whereas fungal community assembly was shaped by both stochastic processes and variable selection. These findings are consistent with studies conducted at moderate spatial scales (Xu et al. 2020; Jiao et al. 2022). Prokaryotes generally exhibit rapid growth and strong sensitivity to nutrient availability, while fungi display greater tolerance to environmental fluctuations (Xu et al. 2020). Consequently, environmental factors in biocrusts promoted convergence among bacterial communities through homogeneous selection, whereas localized selective pressures contributed to fungal community differentiation without serving as the main assembly mechanism.

As the results showed, the pH, nitrogen, soil organic carbon, and phosphorus content were abiotic factors notably associated with the variations in bacterial and fungal communities. These results were consistent with previous studies in biocrusts (Miralles et al. 2020) and grassland soils (Chen et al. 2015). A prominent negative association between nitrogen and the bacterial (excluding cyanobacteria) community was observed, reflecting the high nutrient demand of microorganisms in oligotrophic environments (Eilers et al. 2010; Leff et al. 2015). The effects of nitrogen, SOC, and phosphorus exceeded those of pH, long considered a dominant determinant of microbial communities (Maestre et al. 2015; Fierer 2017). This pattern underscores the central role of nutrient limitation in shaping the microbial community structure of desert biocrusts. Moreover, previous studies have highlighted strong associations between the accumulation of nitrogen or organic carbon and the presence of cyanobacteria (Yeager et al. 2007; Munoz‐Rojas et al. 2018). In sparsely vegetated desert ecosystems, the carbon and nitrogen fixed by cyanobacteria represent major pathways through which autotrophic microorganisms shape biocrust microbial communities (Maier et al. 2018). These previous studies and PLSPM results of this study suggested that cyanobacteria play a role in structuring microbial communities indirectly by regulating physicochemical conditions.

Cyanobacteria‐related interspecies interactions represent another key mechanism influencing microbial community structure. Although cyanobacteria are widely recognized as primary drivers of microbial assemblages in biocrusts (Büdel et al. 2016; Lan et al. 2022), empirical evidence from biocrust systems remains limited. The microbial coexistence plays a crucial role in shaping microbial communities (Stegen et al. 2013). As demonstrated by the network analysis, cyanobacteria had extensive co‐occurrence with *Craurococcus caldovatus*, *Rubellimicrobium*, *Rubrobacter*, and *Microvirga*. These interactions enhanced the robustness and reduced the vulnerability of microbial co‐occurrence networks, particularly during early biocrust stages. Such findings provide insight into potential strategies for stabilizing microbial communities in dryland soils.

### 4.3 Keystone Cyanobacteria in Constructing Stable Microbial Communities

The role of cyanobacteria in microbial community structure is not uniform, as demonstrated in this study. Only a few cyanobacterial species were identified as keystone taxa relative to the overall cyanobacterial richness. These keystone species, primarily affiliated with Chroococcidiopsidaceae, Microcoleaceae, and Nostocaceae, play a disproportionate role in maintaining community stability. Keystone taxa are defined as species that exert a profound influence on the persistence and structure of microbial communities (Banerjee et al. 2018; Rawstern et al. 2025). In algal crusts, where more cyanobacteria were included in the co‐occurrence network, the random removal of all cyanobacterial nodes did not affect network robustness. However, the targeted removal of keystone cyanobacteria caused a significant decline. This result provided direct evidence of functional differentiation among cyanobacteria in shaping microbial communities.

This differentiation may result from variations in the metabolic capacities of cyanobacteria. While cyanobacteria proliferate during the algal crust stage (Zhao et al. 2021), taxa with strong nutrient‐transformation capabilities—such as nitrogen fixation and phosphorus mineralization—typically dominate later successional stages (Gao and Garcia‐Pichel 2011; Xu et al. 2023). The mismatch between nutrient availability and microbial growth demands may therefore drive heterotrophic microbes to assemble around cyanobacteria with high carbon and nitrogen fixation activities. At the later stages of biocrust development, the introduction of mosses replenished resources such as organic matter (Colesie et al. 2012). As a result, the importance of cyanobacteria in maintaining the stability of the microbial community was reduced, as demonstrated by this study. These findings further highlight the significant impact of resource‐fixing microorganisms on the microbial community.

These findings based on data analysis provide valuable insights for biocrust restoration in dryland ecosystems. Traditional cyanobacterial inoculation using pure cultures often faces challenges, including high water requirements during early establishment and limited persistence of the inoculated strains (Bowker et al. 2020; Rossi et al. 2022). Developing synthetic microbial consortia based on keystone cyanobacteria represents a promising alternative. Such consortia can perform complex ecological functions through division of labor and interspecies coordination, enhancing both structural and functional stability (Jagmann and Philipp 2014; Jing et al. 2024). In this study, the putative keystone Chroococcidiopsidaceae and its strong co‐occurrence with *Craurococcus caldovatus*, *Rubellimicrobium*, *Geodermatophilus*, and *Alternaria* provide potential targets for constructing synthetic microbial communities tailored for biocrust restoration. However, this study cannot establish precise insights into the relationship between cyanobacteria and microbial community structure using the correlation‐based method. To confirm causal links, more rigorous experimental designs like manipulative experiments are needed. Unaccounted factors, such as microbial dispersal, could also contribute to the observed patterns. Future research with long‐term monitoring could provide stronger evidence of cyanobacteria's causal role.

## 5 Conclusion

This study investigated cyanobacterial community dynamics and their associations with bacterial and fungal community variations across biocrust succession in a temperate desert. Phosphorus emerged as the primary factor influencing cyanobacterial distribution in biocrusts. The cyanobacterial community, together with the abiotic properties of biocrusts, such as phosphorus, pH, nitrogen, and organic carbon content, explained a significant proportion of the variation in the microbial community. Co‐occurrence network analyses revealed that keystone cyanobacteria, mainly from Chroococcidiopsidaceae, and to a lesser extent Microcoleaceae and Nostocaceae, played a central role in maintaining microbial network stability. These keystone taxa underwent succession alongside biocrust development and exhibited strong associations with *Craurococcus caldovatus*, *Rubellimicrobium*, *Rubrobacter*, and *Microvirga*. Overall, our findings highlight the ecological significance of cyanobacteria in structuring microbial communities and provide a theoretical basis for optimizing biocrust restoration strategies in dryland ecosystems.

## Author Contributions

**Kang Zhao:** conceptualization (equal), data curation (equal), funding acquisition (equal), investigation (equal), supervision (equal), writing – original draft (equal). **Ran Zhao:** investigation (equal), writing – original draft (equal). **Khan Ajmal:** data curation (equal), writing – review and editing (equal). **Wei Chen:** data curation (equal), writing – review and editing (equal). **Qiuping Zhang:** data curation (equal), writing – review and editing (equal). **Bingchang Zhang:** conceptualization (equal), data curation (equal), writing – review and editing (equal). **Fei Wang:** conceptualization (equal), data curation (equal), writing – review and editing (equal).

## Funding

This work was supported by the National Natural Science Foundation of China, 32571921 42271067, and U2003214 Central Guidance for Local Science and Technology Development Funds, YDZJSX20231A052. The Basic Research Program of Shanxi Province 202303021212152 and 202503021211198.

## Ethics Statement

The authors have nothing to report.

## Conflicts of Interest

The authors declare no conflicts of interest.

## Supporting information


**Appendix S1:** Supporting Information.

**Appendix S2:** Supporting Information.

## Data Availability

The physicochemical properties were accessible in the Dryad Digital Repository via the link: https://doi.org/10.5061/dryad.66t1g1k7n. The primary sequence data were archived at the National Center for Biotechnology Information (NCBI) under the accession numbers: PRJNA1048298 (16S) and PRJNA1048333 (ITS).
