# Supplementary material for: Succession of Cyanobacterial Community Contributes to Bacterial and Fungal Community Assembly in Dryland Biocrusts
Source: Ecol Evol. 2026 Mar 2;16(3):e73151. doi: 10.1002/ece3.73151 (PMC12953004; doi:10.1002/ece3.73151)
Supplement: Supplementary file 1 — Appendix S1: Supporting Information. [file ECE3-16-e73151-s001.docx]

**Succession of Cyanobacterial Community Contributes to Bacterial and Fungal Community Assembly in Dryland Biocrusts**

Kang Zhao ^1, 4^, Ran Zhao ^1^, Khan Ajmal ^2^, Wei Chen ^1^, Qiuping Zhang ^1^, Bingchang Zhang ^3*^, Fei Wang ^1*^

^1^ School of Life Sciences, Shanxi Normal University, Taiyuan 030031, China

^2^ Department of Environmental Sciences, Kohat University of Science and Technology, Kohat 26000, Pakistan

^3^ Biodiversity and Ecological Function Research Group of Middle reaches of Yellow River, Geographical Science College, Shanxi Normal University, Taiyuan 030031, China

^4^ Research Center for Ecological Restoration, School of Life Sciences, Shanxi Normal University, Taiyuan 030031, China

Supplementary text 1

The physicochemical properties of the biocrusts are summarized in Table S1. All collected samples were extremely dry and nearly devoid of moisture. The highest pH value (8.14) was recorded in the bare sand biocrust, whereas the lowest (7.84) was observed in the moss crust. Notably, total nitrogen (TN), ammonium, total phosphorus (TP), and soil organic carbon (SOC) levels increased significantly during biocrust development (*p* < 0.05). Specifically, TN increased 4.8-fold (from 0.20 to 0.96 g/kg soil), TP increased 2.1-fold (from 0.11 to 0.24 g/kg soil), and SOC increased 6.5-fold (from 1.78 to 11.72 g/kg soil). Ammonium content also rose progressively, reaching its highest concentration (34.35 mg/kg soil) in the moss crust. Among the biocrust types, the algal crust exhibited the lowest available phosphorus (AP) content (6.53 mg/kg soil), whereas the moss crust showed the highest (7.79 mg/kg soil). Nitrate levels fluctuated throughout the successional stages, with no statistically significant differences detected.

Supplementary text 2

Amplicon sequencing was performed based on the MiSeq platform in paired-end mode (2 × 250 bp), following protocols described in our previous study (Zhao et al., 2024). Low-quality reads were filtered using Trimmomatic v0.33 (SLIDINGWINDOW:50:20) (Bolger et al., 2014). Paired-end reads were merged using USEARCH v10 (Edgar, 2013), and chimeric sequences were removed using the UCHIME algorithm (Edgar et al., 2011). High-quality 16S rDNA sequences were processed using the QIIME2 pipeline (Bolyen et al., 2019), and amplicon sequence variants (ASVs) were obtained. For ITS sequences, operational taxonomic units (OTUs) were clustered at a 97% identity threshold using USEARCH v10. A representative sequence from each OTU or ASV was randomly selected for taxonomic assignment using the SILVA (Quast et al., 2013) and UNITE (Nilsson et al., 2019) databases for bacteria and fungi, respectively. The cyanobacterial community was extracted from the 16S ASV tables. For more accurate phylogenetic resolution, cyanobacterial annotations were manually refined using the NCBI Taxonomy Browser (https://www.ncbi.nlm.nih.gov/Taxonomy/Browser/wwwtax.cgi) at the genus or higher taxonomic levels. Sequence reads were rarefied to 42,536 for 16S rDNA and 56,214 for ITS.

Supplementary text 3

Further analyses were conducted on the co-occurrence patterns of bacteria and fungi during biocrust succession (Fig. S7A and B). The results revealed distinct modular structures, with significant differences in nestedness, weighted nestedness, and modularity indices between empirical and random networks (Table S4). Major network topological properties varied significantly along the biocrust successional gradient (Fig. S7B). Algal and lichen crusts exhibited a higher number of nodes and links, greater clustering coefficients, and higher average node degrees, along with lower modularity, compared to the bare sand and moss crusts. Cyanobacteria in co-occurrence networks were predominantly associated with unclassified Ascomycota, unclassified fungi, *Craurococcus caldovatus*, *Rubellimicrobium*, *Rubrobacter*, and *Microvirga* (Fig. S7C). Putative keystone taxa were identified by classifying network nodes based on their Zi and Pi values (Table S5). The composition of keystone taxa shifted with biocrust development, with only a few groups persisting across multiple stages. Most keystone taxa belonged to the phyla Actinobacteria, Cyanobacteria, Proteobacteria, and Ascomycota.

**References**

Bolger, A M, Lohse, M, Usadel, B, 2014. Trimmomatic: a flexible trimmer for Illumina sequence data. Bioinformatics 30:2114-2120.

Bolyen, E, Rideout, J R, Dillon, M R, Bokulich, N A, Abnet, C C, Al-Ghalith, G A, Alexander, H, Alm, E J, Arumugam, M, Asnicar, F, Bai, Y, Bisanz, J E, Bittinger, K, Brejnrod, A, Brislawn, C J, Brown, C T, Callahan, B J, Caraballo-Rodriguez, A M, Chase, J, Cope, E K, Da Silva, R, Diener, C, Dorrestein, P C, Douglas, G M, Durall, D M, Duvallet, C, Edwardson, C F, Ernst, M, Estaki, M, Fouquier, J, Gauglitz, J M, Gibbons, S M, Gibson, D L, Gonzalez, A, Gorlick, K, Guo, J, Hillmann, B, Holmes, S, Holste, H, Huttenhower, C, Huttley, G A, Janssen, S, Jarmusch, A K, Jiang, L, Kaehler, B D, Bin Kang, K, Keefe, C R, Keim, P, Kelley, S T, Knights, D, Koester, I, Kosciolek, T, Kreps, J, Langille, M G I, Lee, J, Ley, R, Liu, Y-X, Loftfield, E, Lozupone, C, Maher, M, Marotz, C, Martin, B D, McDonald, D, McIver, L J, Melnik, A V, Metcalf, J L, Morgan, S C, Morton, J T, Naimey, A T, Navas-Molina, J A, Nothias, L F, Orchanian, S B, Pearson, T, Peoples, S L, Petras, D, Preuss, M L, Pruesse, E, Rasmussen, L B, Rivers, A, Robeson, M S, II, Rosenthal, P, Segata, N, Shaffer, M, Shiffer, A, Sinha, R, Song, S J, Spear, J R, Swafford, A D, Thompson, L R, Torres, P J, Trinh, P, Tripathi, A, Turnbaugh, P J, Ul-Hasan, S, van der Hooft, J J J, Vargas, F, Vazquez-Baeza, Y, Vogtmann, E, von Hippel, M, Walters, W, Walters, W, Wan, Y, Wang, M, Warren, J, Weber, K C, Williamson, C H D, Willis, A D, Xu, Z Z, Zaneveld, J R, Zhang, Y, Zhu, Q, Knight, R, Caporaso, J G, 2019. Reproducible, interactive, scalable and extensible microbiome data science using QIIME 2 Nature Biotechnology 37:1091-1091.

Edgar, R C, 2013. UPARSE: highly accurate OTU sequences from microbial amplicon reads. Nature Methods 10:996-+.

Edgar, R C, Haas, B J, Clemente, J C, Quince, C, Knight, R, 2011. UCHIME improves sensitivity and speed of chimera detection. Bioinformatics 27:2194-2200.

Feng, K, Peng, X, Zhang, Z, Gu, S, He, Q, Shen, W, Wang, Z, Wang, D, Hu, Q, Li, Y, Wang, S, Deng, Y, 2022. iNAP: An integrated network analysis pipeline for microbiome studies. iMeta 1:e13.

Nilsson, R H, Larsson, K-H, Taylor, A F S, Bengtsson-Palme, J, Jeppesen, T S, Schigel, D, Kennedy, P, Picard, K, Gloeckner, F O, Tedersoo, L, Saar, I, Koljalg, U, Abarenkov, K, 2019. The UNITE database for molecular identification of fungi: handling dark taxa and parallel taxonomic classifications. Nucleic Acids Research 47:D259-D264.

Quast, C, Pruesse, E, Yilmaz, P, Gerken, J, Schweer, T, Yarza, P, Peplies, J, Gloeckner, F O, 2013. The SILVA ribosomal RNA gene database project: improved data processing and web-based tools. Nucleic Acids Research 41:D590-D596.

Zhao, K, Zhang, L, Wang, F, Li, K, Zhang, Y, Zhang, B, 2024. Dynamics in eukaryotic algal communities regulate bacterial and fungal communities as biocrusts develop in a temperate desert in Central Asia. Functional Ecology 38:531-545.

Fig. S1


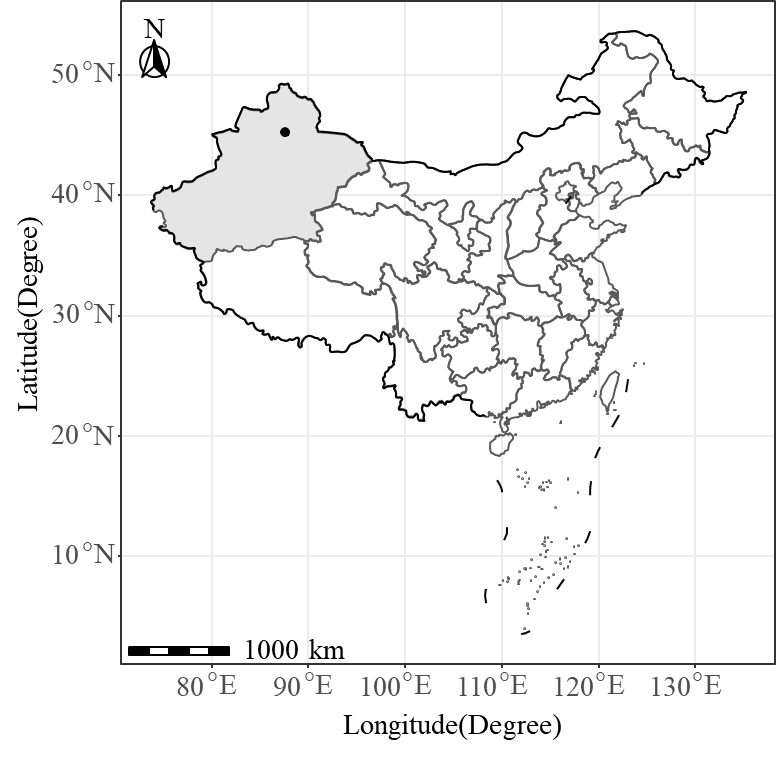


Figure S1 Map of Sampling Point Locations.

Fig. S2


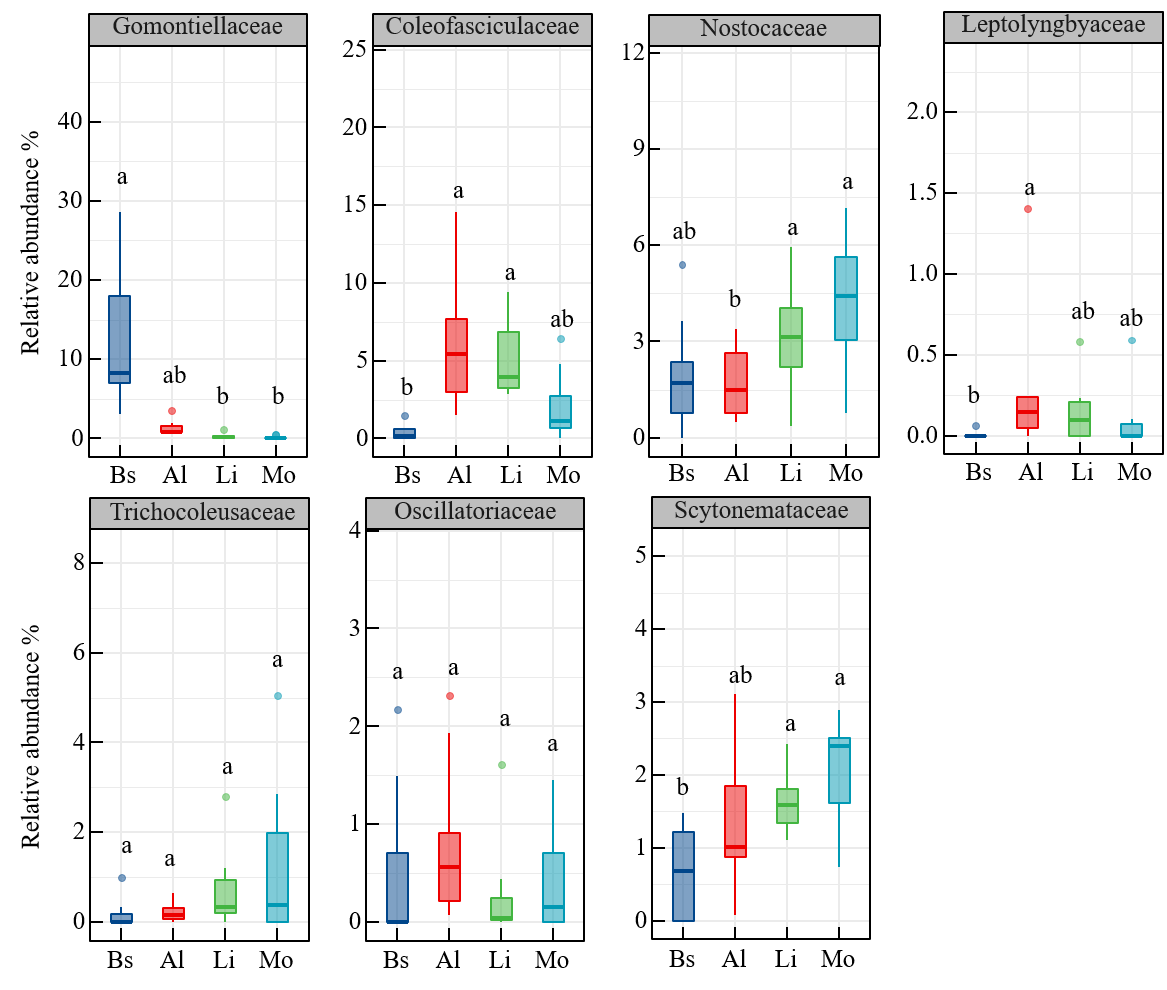


Figure S2 The variational patterns of cyanobacteria in relative abundance at the family level with the succession of biocrust. Bs: bare sand, Al: algal crust, Li: lichen crust, Mo: moss crust.

Fig. S3


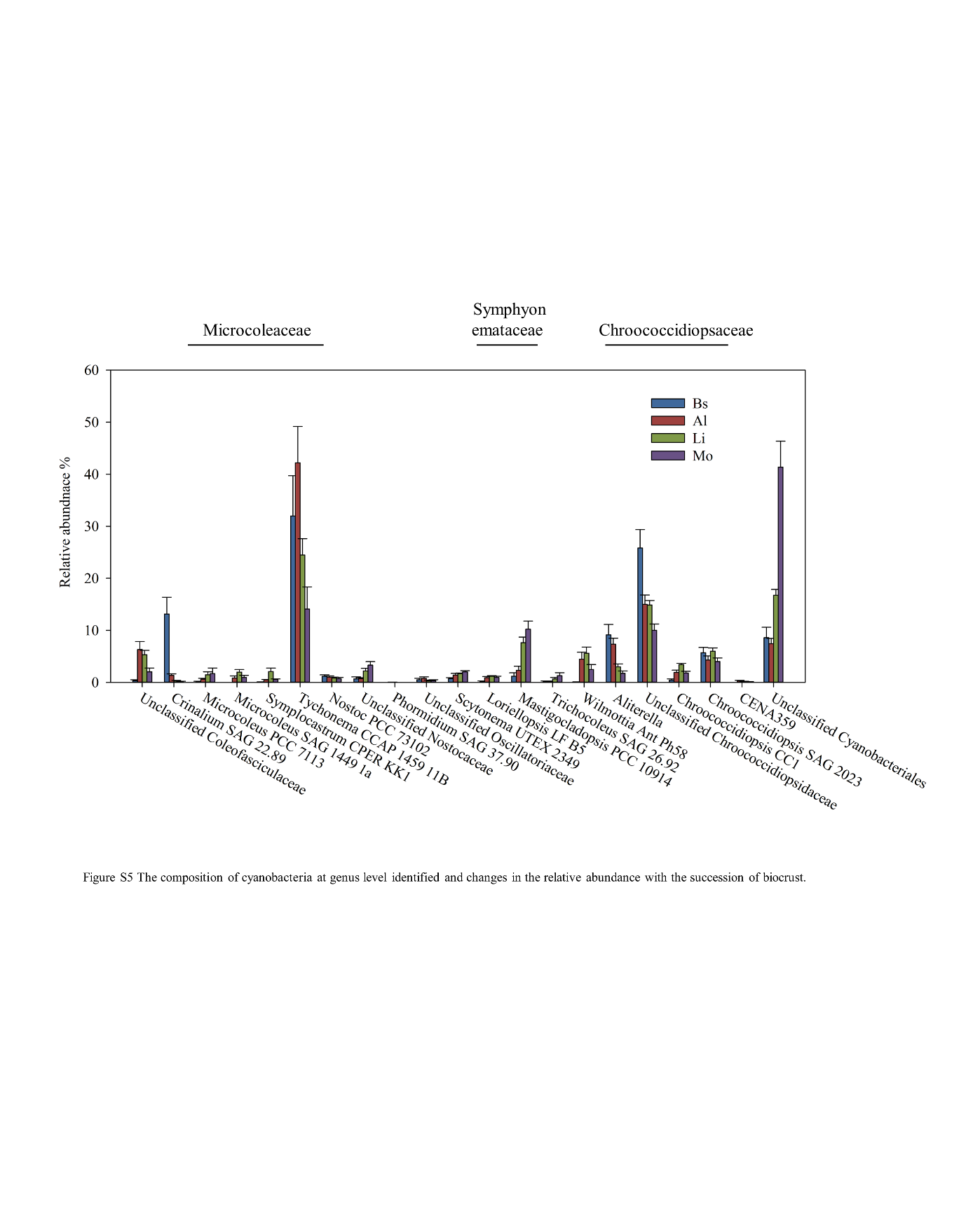


Figure S3 The composition of cyanobacteria at genus level and changes in relative abundance with the succession of biocrust.

Fig. S4


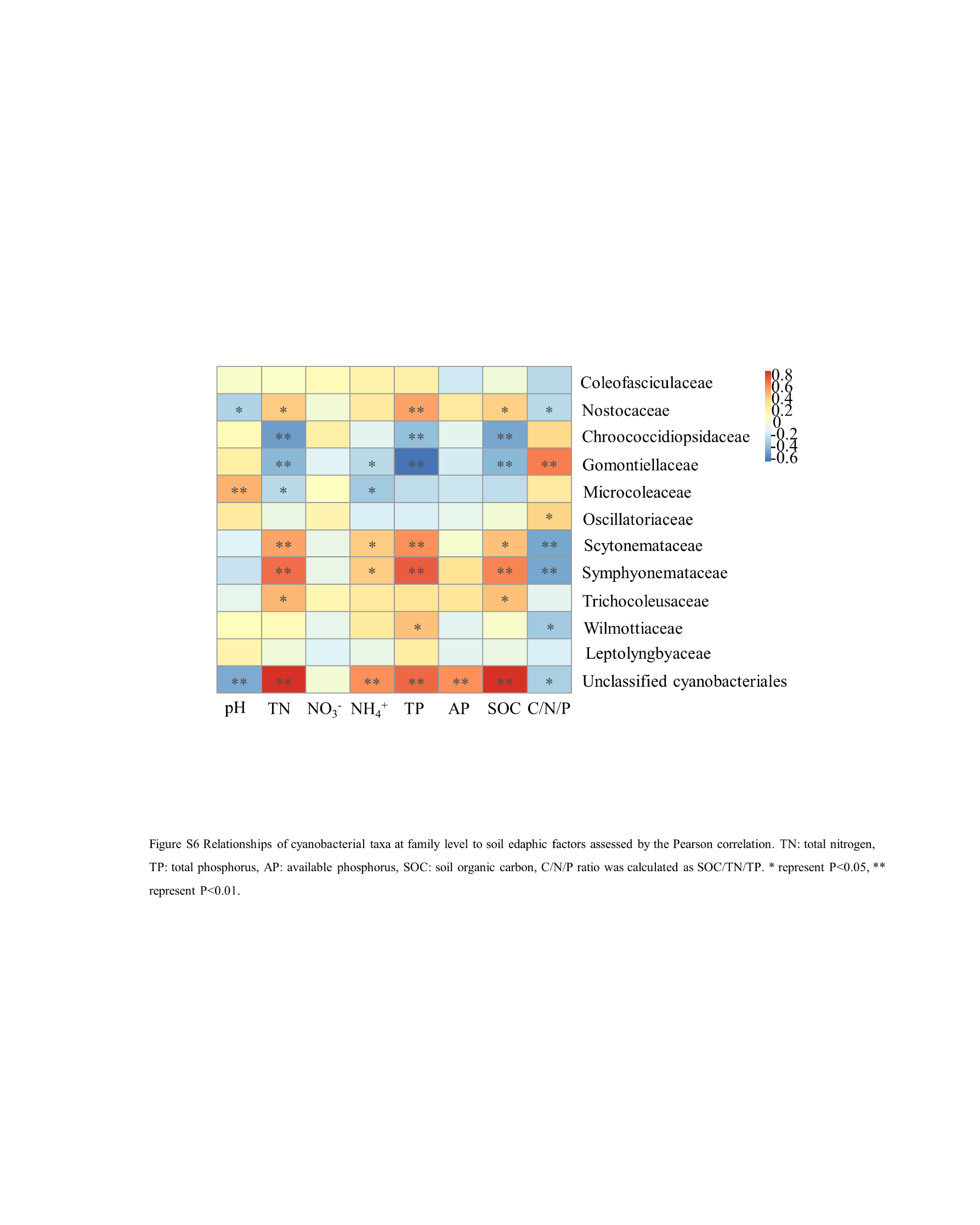


Figure S4 Relationships of cyanobacterial taxa at the family level with soil edaphic factors assessed by Pearson correlation. TN: total nitrogen, TP: total phosphorus, AP: available phosphorus, SOC: soil organic carbon, and C / N / P ratio was calculated as SOC/TN/TP. * represents *p* < 0.05, ** represents *p* < 0.01.

Fig. S5


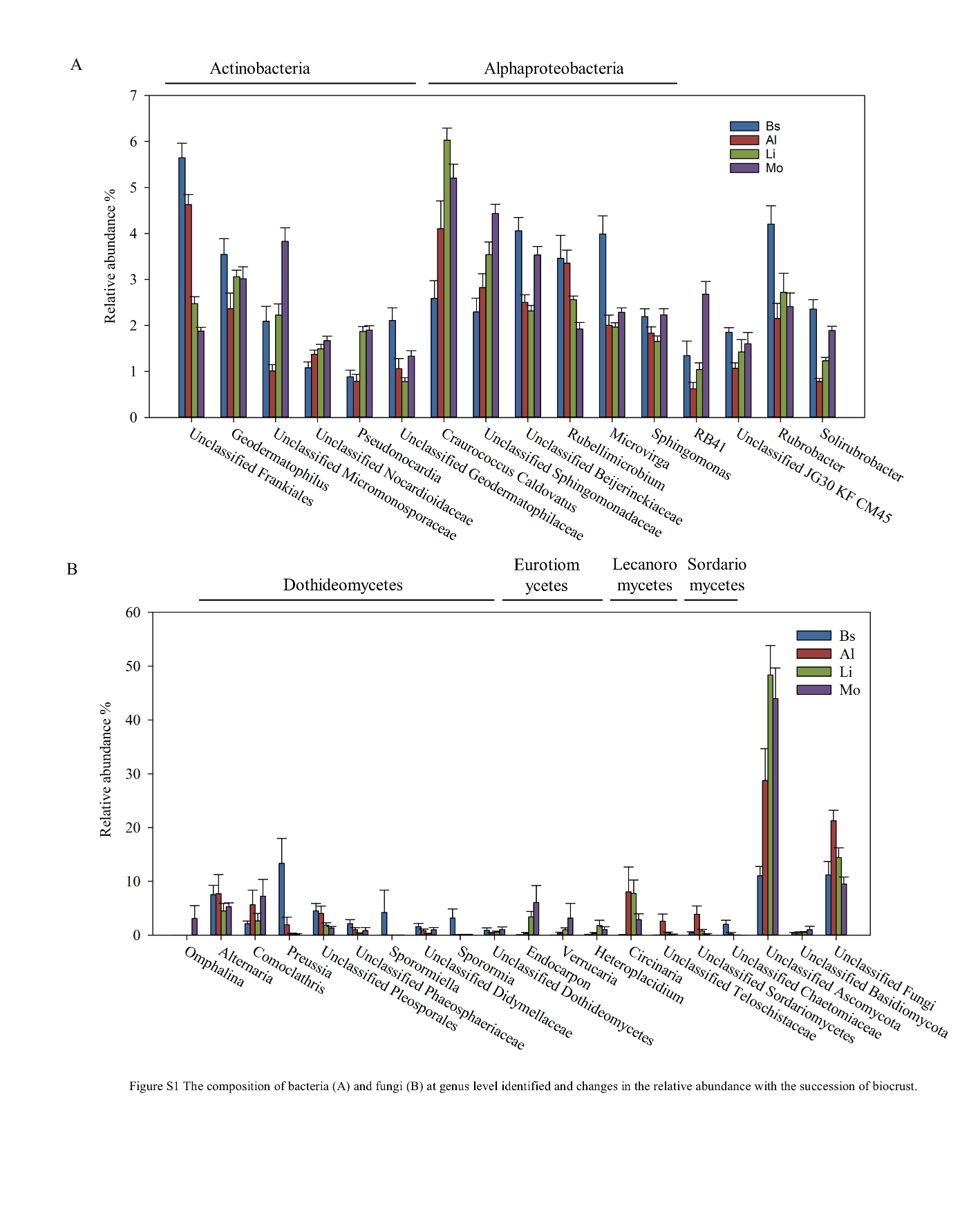


Figure S5 The composition of bacteria (A) and fungi (B) identified at the genus level and changes in relative abundance with succession of biocrust.

Fig. S6


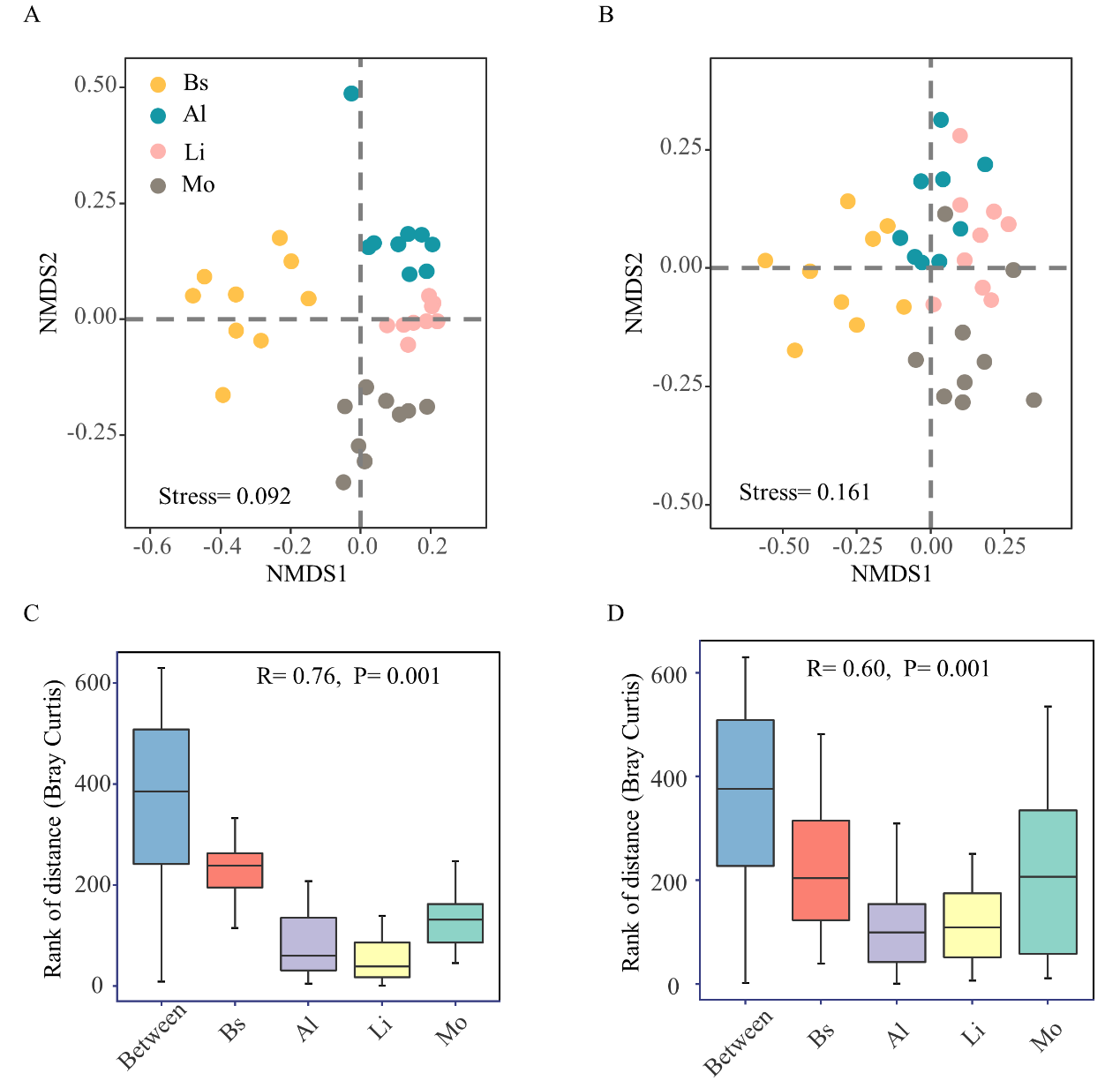


Figure S6 NMDS and ANOSIM results of the bacterial (A and C) and fungal (B and D) community structure

Fig. S7


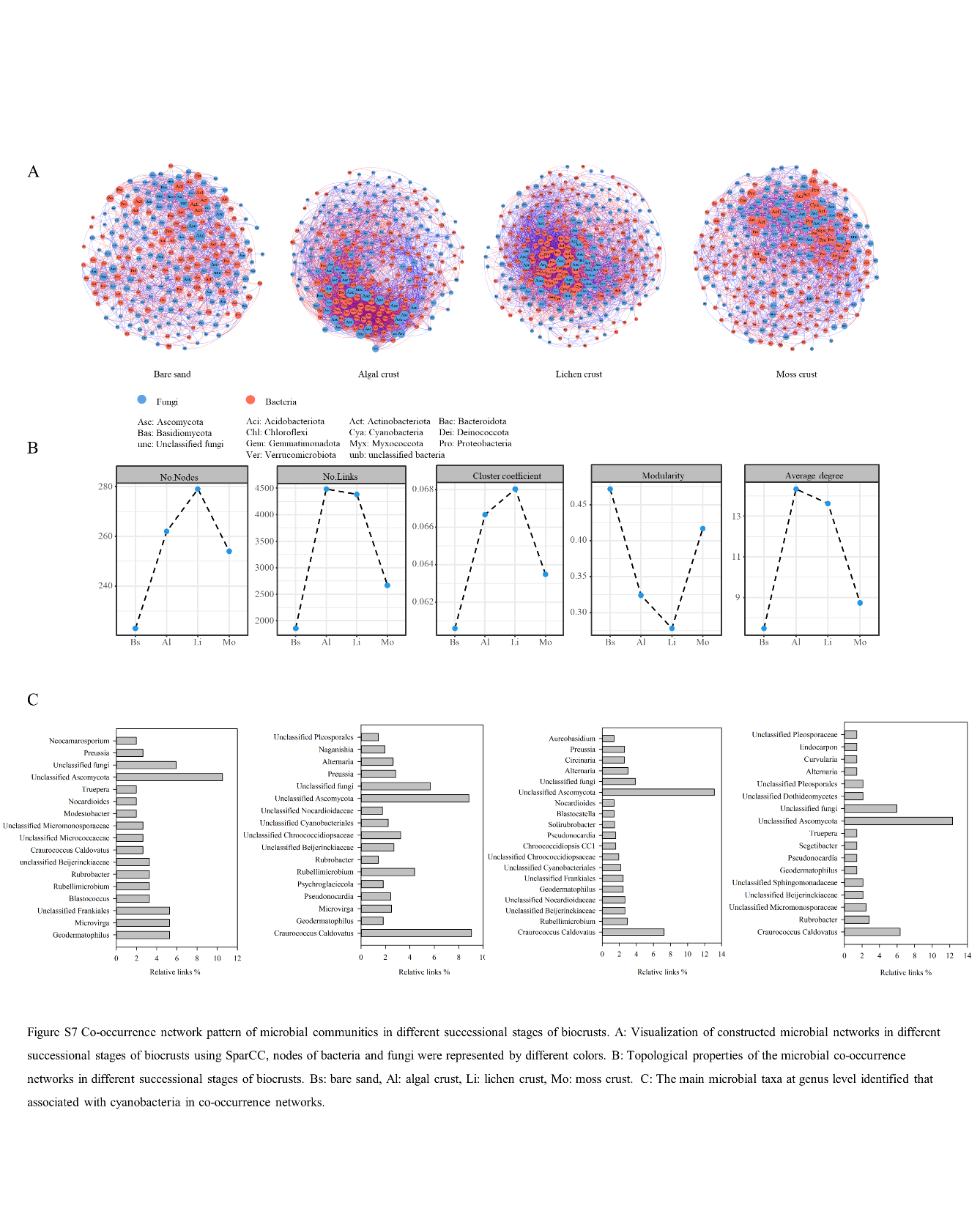


Figure S7 Pattern of co-occurrence networks of microbial communities at different successional stages of biocrust. A: Visualization of constructed microbial networks in different successional stages of biocrusts using SparCC, nodes of bacteria and fungi were represented by different colors. B: Topological properties of the microbial co-occurrence networks in different successional stages of biocrusts. Bs: bare sand, Al: algal crust, Li: lichen crust, Mo: moss crust. C: The main microbial taxa identified associated with cyanobacteria in co-occurrence networks.

Fig. S8


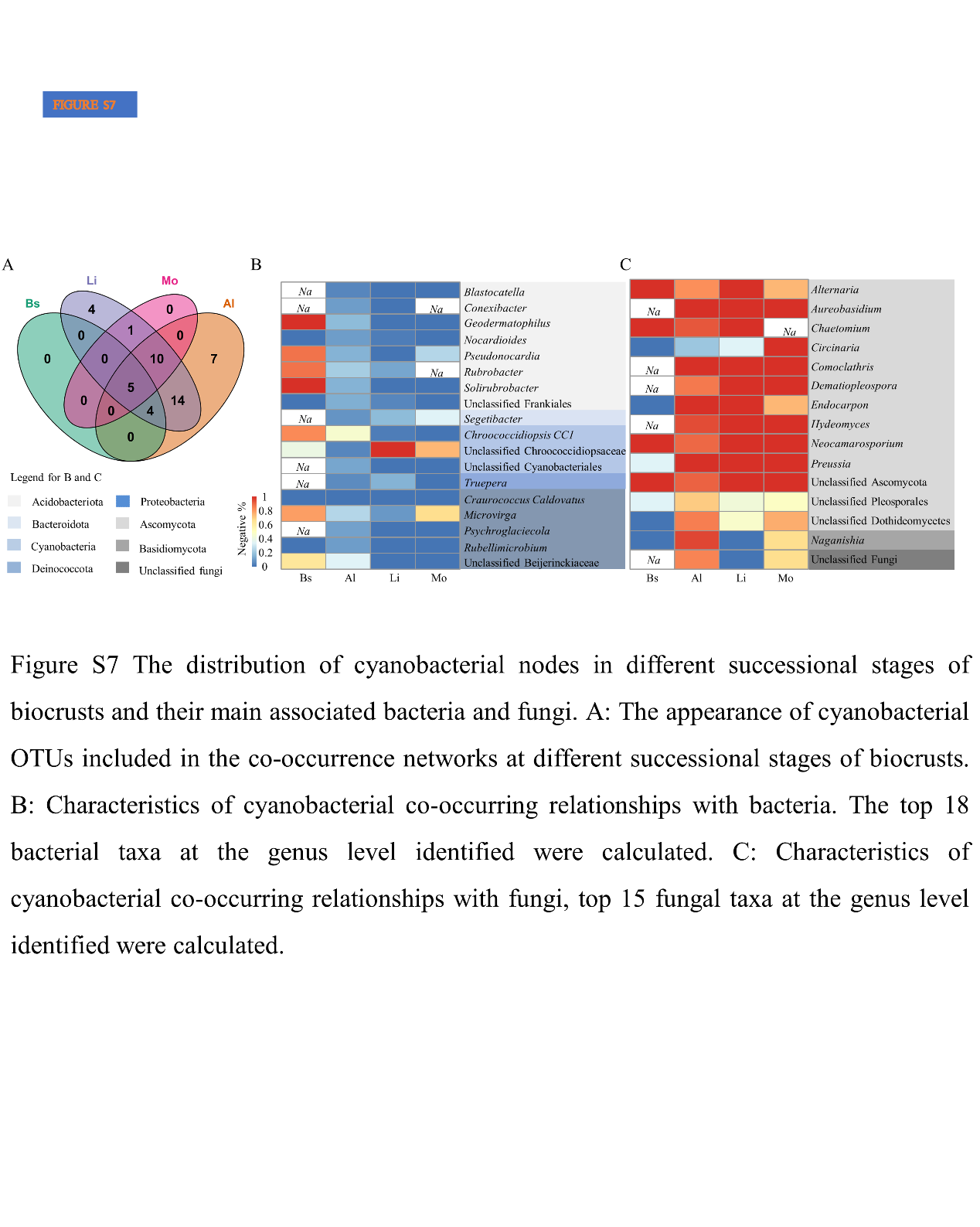


Figure S8 Distribution of cyanobacterial nodes across different successional stage of biocrusts and their primary bacterial and fungal associates. A: The appearance of cyanobacterial ASVs included in the co-occurrence networks at different successional stages of biocrusts. B: Characteristics of cyanobacterial co-occurring relationships with bacteria. The top 18 bacterial taxa at the genus level identified were calculated. C: Characteristics of cyanobacterial co-occurring relationships with fungi, top 15 fungal taxa at the genus level identified were calculated.

Fig. S9


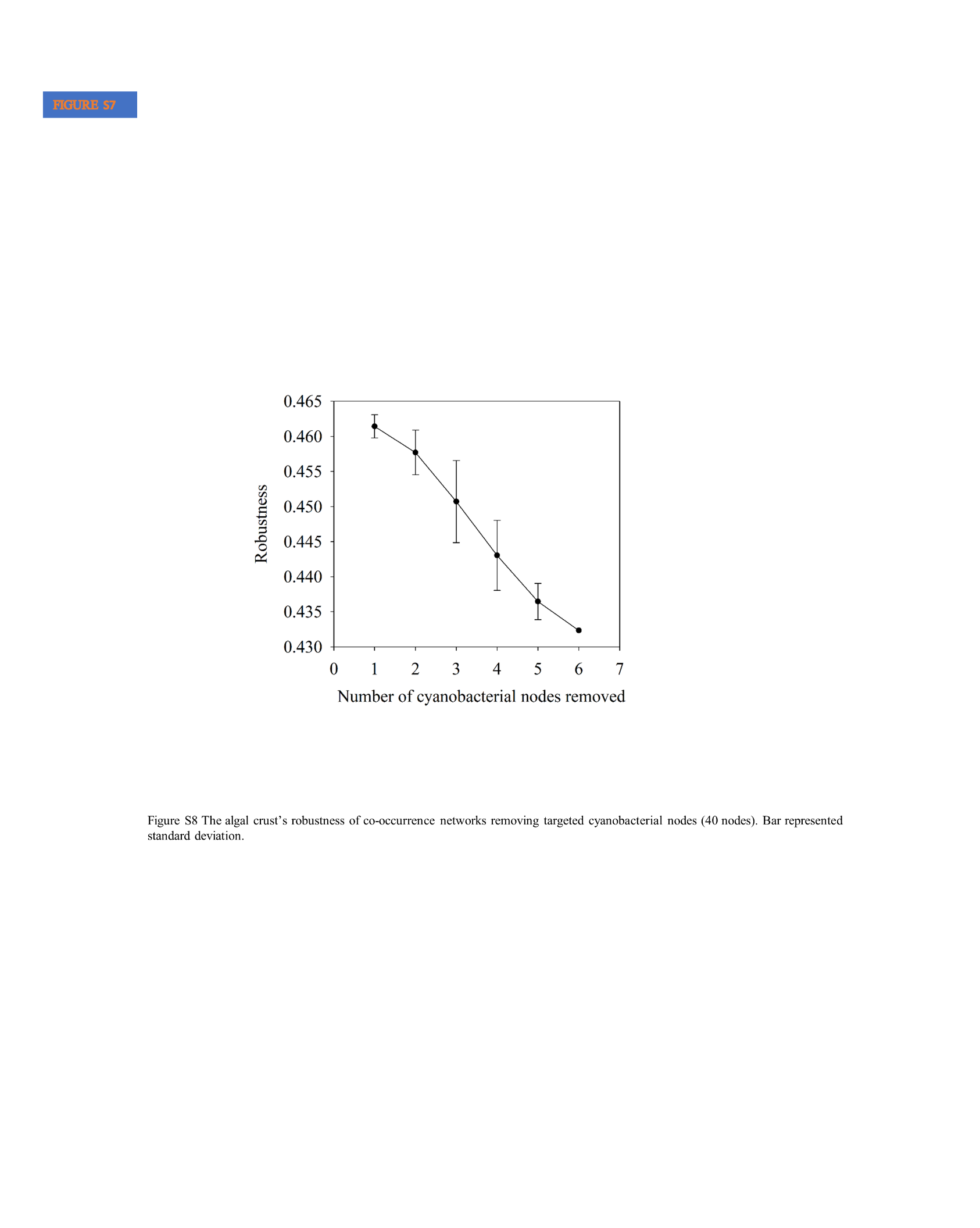


Figure S9 The algal crust’s robustness of co-occurrence networks after removing keystone cyanobacterial nodes. The bar represents the standard error.


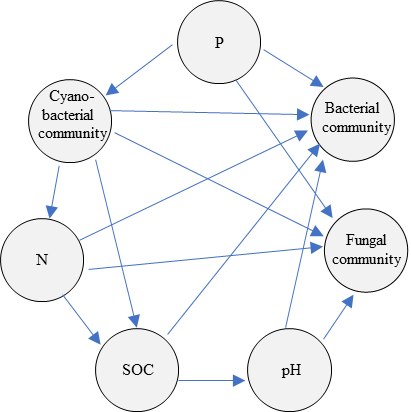


Figure S10 The constructed original model of the PLSPM analysis based on previous studies and prior results of this study.
